# Abnormalities of the external genitalia and groins among primary school boys in Bida, Nigeria

**DOI:** 10.4314/ahs.v17i4.20

**Published:** 2017-12

**Authors:** Adedeji O Adekanye, Samuel A Adefemi, Kayode A Onawola, John A James, Ibrahim T Adeleke, Mark Francis, Ezekiel U Sheshi, Moses E Atakere, Abdullahi D Jibril

**Affiliations:** 1 Centre for Health & Allied Researches (CHAR), Federal Medical Centre Bida, Nigeria; 2 Department of Surgery, Federal Medical centre, Bida Nigeria; 3 Department of Family Medicine, Federal Medical centre, Bida Nigeria; 4 Department of Health Information management, Federal Medical centre, Bida Nigeria; 5 Department of Obstetrics & Gynaecology, Federal Medical centre, Bida Nigeria

**Keywords:** External genitalia, groin abnormalities, boys

## Abstract

**Background:**

Abnormalities of the male external genitalia and groin, a set of lesions which may be congenital or acquired, are rather obscured to many kids and their parents and Nigerian health care system has no formal program to detect them.

**Objectives:**

To identify and determine the prevalence of abnormalities of external genitalia and groin among primary school boys in Bida, Nigeria.

**Methods:**

This was a cross-sectional study of primary school male pupils in Bida. A detailed clinical examination of the external genitalia and groin was performed on them.

**Results:**

Abnormalities were detected in 240 (36.20%) of the 663 boys, with 35 (5.28%) having more than one abnormality. The three most prevalent abnormalities were penile chordee (37, 5.58%), excessive removal of penile skin (37, 5.58%) and retractile testis (34, 5.13%). The prevalence of complications of circumcision was 15.40% and included excessive residual foreskin, excessive removal of skin, skin bridges and meatal stenosis. Undescended testes were seen in 6 (0.90%) boys, with median age of 9 years and 2 were bilateral. Also, micropenis was detected in 27 (4.07%) of the pupils.

**Conclusion:**

Inguino-penoscrotal abnormalities are common in our community (36.20%). Screening of pre-school and school children to detect them should be introduced into the school health programs in Nigeria.

## Introduction

Abnormalities of the male external genitalia and groins are a set of lesions which may be congenital or acquired but are rather obscured to many kids and their parents. This is particularly so where the parents may not be certain of what a normal external genitalia and groin should look like, or out of sheer negligence. The absence of any immediate functional derangement usually reinforces such indifference to the presence of the abnormalities. Late presentation may then be inevitable, which may be in form of life-threatening complications such as strangulated hernia or grave sequelae such as infertility, which is not uncommon in the practice of the lead author (a Urologist). The optimal time for the treatment of most of these surgically correctable lesions is infancy and childhood.

Among the congenital ones are hypospadias, epispadias, buried penis, micropenis, ambiguous genitalia, phimosis, paraphimosis, aphallia (penile agenesis), diphallia, chordee without hypospadia, hernia, hydrocoele, bifid scrotum, ectopic scrotum, cryptorchidism/undescended testis, retractile testes, epididymal cyst, etc.

The acquired ones may result from circumcision which includes excessive residual foreskin, excessive removal of skin, meatal stenosis, granuloma, penile torsion, secondary chordee, skin bridges, glans amputation, inclusion cyst, urethro-cutaneous fistula etc.

Previous studies on these abnormalities reported prevalence as low as 6.65% and as high as 18.31%, among primary school male pupils aged 6–12 years.^1^,^2^

In Nigeria, there is no formal setup or program in our health care system for the detection of these abnormalities. The observed pattern of presentation of patients with these abnormalities at our health facility is that of late presentation in adulthood, presentation with complications and grave sequelae, incidental detection of the lesions during treatment of other conditions, etc; Therefore, the present study sought to identify congenital and acquired abnormalities of male external genitalia and groin among primary school pupils in Bida, and determine their prevalence.

## Methods

This was a cross-sectional study carried out among male primary school pupils in Bida, North Central Nigeria. By systematic random sampling, 10 were selected out of the 75 primary schools in Bida.

Ethical approval was obtained from the “Ethics Committee” (IRB) of the Hospital for the study. Approval from the management of the selected schools was also obtained for the study to be carried out among its pupils. Eight of the 10 schools gave the approval, representing 10.6% of all the schools in the community. All male pupils in the selected schools were included. Informed consent was obtained from each male pupil's parents before data collection and examination of the external genitalia and groin of their wards. The parents were also informed of the availability of Specialists in our hospital who could handle the treatment of those with anomalies.

A detailed clinical examination of the external genitalia and groin of the male pupils was done. The examination of the boys was performed by the two urologists and other physicians among the authors. These physicians who are trainee surgeons learned the methods of examination from the urologists over a few pilot sessions. Also, positive or suspicious cases were re-examined by the urologists for confirmation. Findings were recorded on data forms for subsequent data input and analysis, using SPSS version 17. In all, a total of 966 boys' parents were given the consent forms but 663 (68.6 %) boys were included in the study and these were the ones whose parents gave the consent and had complete data for analysis.

## Results

Most of the boys (90 %) were ≤13years, with a mean age of 9.4 ± 3.0 years. Abnormalities were detected in 240 (36.20%) of the 663 boys, with 35 (5.28%) having more than one abnormality. The three most prevalent abnormalities were penile chordee (37, 5.58%), excessive removal of penile skin (37, 5.58%) and retractile testis (34, 5.13%) [Table 1].

**Table 1:** Inguino-scrotal and penile abnormalities among Bida school boys

Abnormalities		Frequency	% Frequency	Mean Age(Min;Max) - yrs
**Inguinoscrotal**	Undescended testis	6	0.90	8.17 (4;10)
	Retractile testis	34	5.13	8.47 (5;14)
	Inguinal hernia	9	1.36	11.33 (8;18)
	Hydrocoele	9	1.36	10.22 (7;16)
	Varicocoele	4	0.60	13.25 (10;15)
	Epididymal cyst	1	0.15	14.00
	Groin scars	2	0.30	9.50 (9;10)
	
**Penile**	Hypospadia	3	0.45	7.67 (5;10)
	Chordee	37	5.58	10.59 (5;15)
	Torsion	43	6.49	10.07 (4;16)
	Excessive residual skin	29	4.37	8.83 (3;15)
	Excessive removal of skin	37	5.58	8.49 (4;17)
	Skin bridges	19	2.87	10.00 (6;15)
	Meatal stenosis	17	2.56	9.65 (4;14)
	Micropenis	27	4.07	8.67 (3;17)

Min – Minimum Max – Maximum Yrs – Years

**Figure 1:**
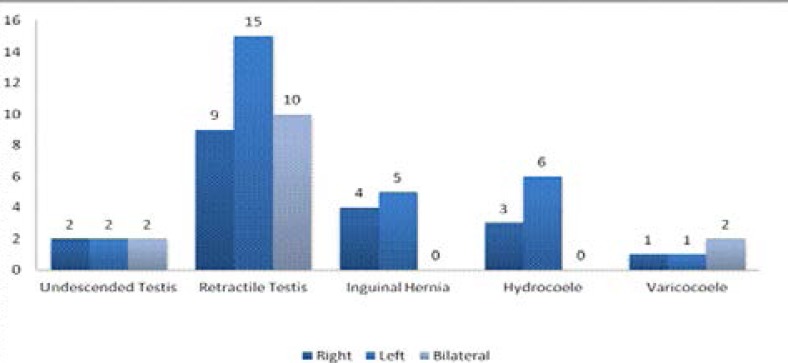
The side involved in inguino-scrotal abnormalities

Of the 34 boys with retractile testis, 9 (26.50%) were on the right, 15 (44.12%) on the left and 10 (29.41%) were bilateral [Fig. 1]. Hydrocoele was detected in 9 (1.36%) of the 663 boys, 3 (33.33%) of the 9 were on the right side and 6 (66.67%) on the left side, with 1 (11.11%) of the latter being an encysted hydrocoele [Table 1, Fig.1]. Undescended testes were seen in 6 (0.90%) boys, with median age of 9 years (range of 4 – 10) and 2 were bilateral. Varicocoele was seen in 4 (0.60%) boys, 1 on the right, 1 on the left and 2 were bilateral [Fig.1]. Indirect inguinal hernia was detected in 9 (1.36%) of 663 boys, 4 on the right side and 5 on the left [Fig.1]. The only case of epididymal cyst (0.15%) was detected on the left side. Our findings revealed 3 (0.45%) of the 663 boys had hypospadia, 37 (5.58%) had chordee, [Right lateral-7, Left lateral-24, Ventral-1, Dorsal-2, 3 not stated] and 43 (6.49%) had penile torsion [24 with <45 degree, 10 with 45–90 degree and 1 with > 90 degree of torsion, 8 with no measurement]. About two-thirds (67.50%) of the 43 boys with torsion were in the anti-clockwise direction. Micropenis was detected in 27 boys (4.07%).

Abnormalities/complications of circumcision found include excessive residual foreskin in 29 (4.37%), excessive removal of penile skin in 37 (5.58%), skin bridges in 19 (2.87%) and meatal stenosis in 17 (2.56%) of the 663 boys. In all, the prevalence of circumcision complications among our study population was 15.40%. This assumes that meatal stenosis is almost always a complication of circumcision.

Other penile abnormalities included penile skin wart/tag in 2 (0.3%) boys. Rather gross congenital penile abnormalities such as ambiguous genitalia, buried penis, apenia, diphalia and epispadia were not seen in our study population. Clinically acute conditions like phimosis and paraphimosis were also not seen.

There were 6 (0.91%) boys with intact prepuce, with mean age of 7years and range of 4 to 10 years. The mean age for each of the group of boys with each abnormality was over 7 years as in table 1.

## Discussion

Inguinoscrotal and penile anomalies, congenital or acquired, are among the common lesions in children and some have no gross functional or morphological import on the patient. But many of them can be corrected by surgical operation at the earliest possible period, preferably when less than 2–3 years of age. This will forestall complications that can be fatal or grave and irreversible. In the practice of the lead author, a number of adult male patients have presented at the clinic with infertility and examination revealed bilateral undescended testis, which as expected were atrophic and located in the groin or abdomen.

The prevalence of undescended testis in this study was found to be 0.90%, which is similar to the widely quoted prevalence of 1% by age 1 and throughout adulthood.^3^ Yegane et al found prevalence of 1.12% in Iran and it was 2.12% in a study from Southern Jordan.^1^,^2^ Different studies from other parts of the globe have demonstrated that the prevalence of undescended testis in boys more than 1 year old is approximately 0.8%.^4^–^7^ Onuora et al also reported a prevalence of 0.5% out of 2200 between the ages of 6 and 12 years in Nigeria.^8^ These boys with undescended testis, particularly with bilateral lesion, may likely have fertility problem later in life, even if the lesion is corrected at their present age. The prevalence of retractile testis in this study was 5.13%. Yücesan et al found the prevalence of 4.45/1000 boys among 19750 Turkish students between the ages of 6years to 15years.^9^ In Southern Jordan, it was 1.26%.^2^ Though the prevalence in our study is rather high, it is not worrisome since retractile testis is usually not considered to have significant functional or morphological implication.

Varicocoele is an anomaly that is rare in boys up to 10 years of age and was found in 16.2% in those 10 – 19 years old.^3^ The prevalence of varicocoele was 0.60% (4 boys) in this study and 2 of them were less than 10 years (6 and 8 years). Zivković et al found the prevalence of varicocoele in boys aged 15 years to be 15.8% out of 1229 elementary School boys that were examined.^10^ Hsieh et al found that 1.02% (22boys) out of 2149 elementary School boys in Taiwan had varicocoele.^11^ This low prevalence is similar to the finding in our study. The prevalence of isolated (non-communicating) hydrocoele in children older than 1 year of age is probably less than 1%.^1^ The prevalence of hydrocoele in this study was 1.36% which was higher than 0.23% found in Southern Jordan and 0.78% found in West of Iran.^1^,^2^ The high prevalence of hydrocoele of 14.2% reported in neighbouring Ghana was among children 5 years and below and this could explain the difference.^12^

In our study, the incidence of indirect inguinal hernia was 1.36%. This is low compared to other studies with figures of between 2.4–13.7% but comparable to that in the general population of between 1–5%.^2^,^10^,^13^,^14^,^15^ The reasons for this could not be proffered based on the design of the study perhaps some other unidentified factors may be responsible. It has however been reported in studies with low incidence that prematurity and other diseases may make the prevalence high which is likely to be absent in the population sampled. As opposed to some studies, we did not use Valsalva maneouver which is likely to increase the yield.^1^,^2^ Also, the low incidence of inguinal hernia, just like for hydrocoele, could not be accounted for by the presence of groin scars (only 2 as in Table 1) which excludes the possibility of having had operations to correct the lesion.

Furthermore, in our study inguinal hernia on the right side was lower than on the left contrary to that reported in the other literature.^2^,^10^,^13^,^14^,^16^ However, it was observed that most of the inguinoscrotal lesions in this study were more on the left than on the right. (see figure 1)

Direct inguinal hernia and femoral hernias in children have been said to be extremely rare and were not found in this study.^15^

Hypospadia is one of the most common malformations of male genitalia with a traditionally quoted incidence of 1:300 male births. However epidemiological evidence suggests that in developed western countries, the incidence is increasing and may be as high as 8 in 1000 (1:125) male births.^17^ The prevalence of hypospadia in our study is about 1 in every 200 which is in conformity with the commonly quoted prevalence of 1 in 250 males.^18^ It is less than 1%, which is similar to the findings in studies in the middle East.^1^,^2^ The few ones seen were glanular or sub-coronal types, which have less conspicuous immediate morphological and/or functional impact on the children. However, all of them with hypospadia have been circumcised, suggesting that the “circumcisers” were unlikely to be medically trained and/or were ignorant of the import of such on the subsequent management of the abnormality. Hypospadia of the distal portion of the penis are many times however covered with a normal foreskin and circumcision can safely be performed in such cases without concern for complication of subsequent repair of the hypospadia.^19^

There was no epispadia, apenia/aphallia nor ambiguous genitalia. This finding corroborates the rarity of these congenital anomalies as similar studies either also had no child or less than 1 in 1,000 of them with any of the abnormalities.^1^,^2^ However, epispadia is quoted to occur in 1 in every 250 males but not finding a single case in our study might be due to the poor management outcome for such cases in our environment with the attendant high mortality.^18^

A review of complications of circumcision by Weiss et al shows that they are rare, but there is wide variation in reported frequencies of these complications.^20^ Identified factors include age at circumcision, training and expertise of the provider, the sterility of the conditions under which the procedure is undertaken and the indication (medical/cultural) for circumcision. Other factors for the variation are methodological issues such as duration of follow-up, epidemiological study design, and definition of complications. The review reported the frequency of complication of circumcision ranging from 0–16%. The review excluded, where possible, inadequate or excessive removal of foreskin as they were considered though arguably, non-medical risks.

This study however found a circumcision complication rate of 15.40%, including inadequate or excessive removal of foreskin as well as meatal stenosis as abnormalities of the external genitalia. This prevalence is similar to that found by Okeke et al in a study on the epidemiology of complications of circumcision in Ibadan, SouthWest Nigeria in which 65 of the 370 (17.57%) children had complications.^21^ High complication rate is associated with circumcision; by inexperienced and/or unskilled provider, in non-sterile settings, with inadequate equipment and at later age groups above neonatal period.^20^,^21^ All these factors are obtainable in the practice of circumcision in our community.

About 1% of the boys in this study were yet to be circumcised. This is quite uncommon in the cultural setting of Nigeria where most boys are circumcised during infancy. On the overall, the prevalence of inguino-penoscrotal abnormalities in this study was 36.20%, which is high. In the study done in the West of Iran, abnormalities were detected in 213 children out of the 3,205 (6.64%) school boys while in the Jordan study, abnormalities of the groin and genitalia were detected in 320 children out of the 1748 (18.31%) boys.^1^,^2^

Most of the parents in some studies were not aware of their children's anomalies.^1^,^2^ In our experience in this study, many parents (31.4%) and 2 of the school authorities contacted did not consent to the study in spite of the detail explanations of the benefits and “no-harm” ethical status of the study. Was it a situation of “ignorance is a bliss” and the parents desiring to maintain that status? That question could not be answered in this study. It is therefore possible that more abnormalities may have been detected if more parents had consented and their wards included. It was obvious that among these parents, the knowledge and appreciation of the health benefits of medical check-up/screening and consequent early detection of health problems were inadequate.

In conclusion, inguino-penoscrotal abnormalities were common in our community (36.20%). Our findings in this study also indicated delay in presentation and diagnosis of inguino-penoscrotal lesions among pupils in our community. Deliberate efforts at screening pre-school and school children are important to avoid future fatal and grave complications. This can be achieved by introducing routine genital and groin examinations into the school and child health programs/services in Nigeria. Targeted health education of the populace, particularly mothers and periodical reminder of health officials on the benefits of early detection and management of these inguino-penoscrotal abnormalities are desirable in our community. These measures will ensure a better outcome for the conditions.

## References

[1] Yegane RA, Kheirollahi AR, Bashashati M (2005). The prevalence of penoscrotal abnormalities and inguinal hernia in elementary school boys in the west of Iran. Int J Urol.

[2] Al-Abbadi K, Smadi SA (2000). Genital abnormalities and groin hernias in elementary school children in Aqaba: an epidemiological study. East Mediterr Health J.

[3] Schneck FX, Bellinger MF Abnormalities of the testes and scrotum and their surgical management Chapter 127 Wein: Campbell-Walsh Urology.

[4] Haji-Nasrollah E (1996). Epidemiology of UDT in students of Tehran. Pajoohesh Dar Pezeshki.

[5] Fonkalsurd WE, Welsh KJ, Randolph MM (1986). Undescended testicle. Pediatric Surgery.

[6] Moul JW, Belman AB (1988). A review of surgical treatment of undescended testes with emphasis on anatomical position. J Urol.

[7] McAninch JW, Tanagho EA, McAninch JW (2000). Disorders of the testis, scrotum and spermatic cord.

[8] Onuora VC, Evbuomwan I (1989). Abnormal findings associated with undescended testis in Nigerian children. Indian J Pediatr.

[9] Yücesan S, Dindar H, Olcay I (1993). Prevalence of congenital abnormalities in Turkish school children. Eur J Epidemiol.

[10] Zivković D, Varga J, Grebeldinger S (2004). [External genital abnormalities in male school children: an epidemiological study]. Med Pregl.

[11] Hsieh TF, Chang CH, Chang SS (2006). Foreskin development before adolescence in 2149 schoolboys. Int J Urol.

[12] Abantanga FA (2003). Groin and scrotal swellings in children aged 5 years and below: a review of 535 cases. Pediatr Surg Int.

[13] Shahram G, Faramarz F, Majid AS (2014). Prevalence of inguinal hernias and genital abnormalities among elementary school boys. Zahedan J Res Med Sci.

[14] Al-Shawawreh A, Abu-Mayyaleh I (2010). Abnormalities of external genitalia and groin hernias in the city of Karak in the south of Jordan. Acta Medica Mediana.

[15] Lugo-Vicente H (2003). Recurrent inguinal hernias. Paed Surg Update.

[16] Tsai YC, Wua CC, Ho CH (2011). Minilaparascopic herniorrhaphy in paediatric inguinal hernia: a durable alternative treatment to standard herniotomy. J Paed Surg.

[17] Paoluzzi L J (2000). Is hypospadia an ‘environmental’ birth defect?. Dialogues Paed Urol.

[18] Elder JS Abnormalities of the genitalia in boys and their surgical management. Chapter 126 Wein: Campbell-Walsh Urology.

[19] Snodgrass WT, Khavari R (2006). Prior circumcision does not complicate repair of hypospadias with an intact prepuce. J Urol.

[20] Weiss HA, Larke N, Halperin D, Schenker I (2010). Complications of circumcision in male neonates, infants and children: a systematic review. BMC Urology.

[21] Okeke LI, Asinobi AA, Ikuerowo OS (2006). Epidemiology of complications of male circumcision in Ibadan, Nigeria. BMC Urology.

